# Architecture of *Paradiplozoon homoion*: A diplozoid monogenean exhibiting highly-developed equipment for ectoparasitism

**DOI:** 10.1371/journal.pone.0192285

**Published:** 2018-02-07

**Authors:** Iveta Hodová, Radim Sonnek, Milan Gelnar, Andrea Valigurová

**Affiliations:** Department of Botany and Zoology, Faculty of Science, Masaryk University, Kotlářská 2, Brno, Czech Republic; Institut de Genetique et Developpement de Rennes, FRANCE

## Abstract

Diplozoidae (Monogenea) are blood-feeding freshwater fish gill ectoparasites with extraordinary body architecture and a unique sexual behaviour in which two larval worms fuse and transform into one functioning individual. In this study, we describe the body organisation of *Paradiplozoon homoion* adult stage using a combined approach of confocal laser scanning and electron microscopy, with emphasis on the forebody and hindbody. Special attention is given to structures involved in functional adaptation to ectoparasitism, i.e. host searching, attachment and feeding/metabolism. Our observations indicate clear adaptations for blood sucking, with a well-innervated mouth opening surrounded by sensory structures, prominent muscular buccal suckers and a pharynx. The buccal cavity surface is covered with numerous tegumentary digitations that increase the area in contact with host tissue and, subsequently, with its blood. The buccal suckers and the well-innervated haptor (with sclerotised clamps controlled by noticeable musculature) cooperate in attaching to and moving over the host. Putative gland cells accumulate in the region of apical circular structures, pharynx area and in the haptor middle region. Paired club-shaped sacs lying laterally to the pharynx might serve as secretory reservoirs. Furthermore, we were able to visualise the body wall musculature, including peripheral innervation, the distribution of uniciliated sensory structures essential for reception of external environmental information, and flame cells involved in excretion. Our results confirm in detail that *P*. *homoion* displays a range of sophisticated adaptations to an ectoparasitic life style, characteristic for diplozoid monogeneans.

## Introduction

Monogenea Bychowsky 1937 are among the most species-rich groups of fish parasites [1]. Monogenean parasites display a direct life cycle, lacking alternation of generations or hosts. Host specificity in the group is well defined, with morphological adaptations to the attachment organs often restricting species to a particular host and/or a very narrow niche [2]. Blood-feeding freshwater fish gill ectoparasites of the family Diplozoidae occupy a unique position amongst monogenean taxa as they exhibit extraordinary body morphology and have a life cycle involving permanent fusion of two larval worms that subsequently transform into a single individual. As such, they represent an attractive model for evolutionary and morphological studies. The first morphological studies on diplozoids were published more than 120 years ago [3–5]. To date, the extensive work of Bovet [6] and Khotenovsky [7] still represents the most comprehensive morphological and taxonomical studies of diplozoid monogeneans. More recent reviews provide useful information on general and functional morphology of monogeneans [8,9]. Numerous studies have already targeted their life cycle and pairing process [10–17], while the other focused on molecular biological [18–22] and karyological [23,24] analyses of representatives from the family Diplozoidae. On the top of that, few immunomicroscopical observations of the diplozoid nervous system were published [14,25,26]. Recent biochemical analyses deal with the blood digestion in diplozoids [27,28].

*Paradiplozoon homoion* is a generalist diplozoid species parasitising a number of cyprinid fish and, as such, represents a suitable model parasite for a range of studies. To date, most studies have concentrated on *Paradiplozoon* spp. genetic characterisation and identification, its life cycle under experimental conditions [29], abnormalities in the attachment apparatus and fluctuating asymmetry [30–32], morphology of the digestive tract [33] and excretory system [34], ultrastructure of the tegument and attachment structures [35]. However, only few fluorescent or methodical studies focusing on *Paradiplozoon* spp. were published to date [36–38]. A recent study visualised the trace element accumulation sites in *Paradiplozoon* adults [39].

Though molecular and biochemical studies are becoming increasingly prevalent, routine microscopic methods, such as electron microscopy and confocal laser scanning microscopy, in combination with immunohistochemistry, still provide a strong tool for investigating different aspects of a parasite’s biology, including its functional morphology and any adaptive mechanisms. A number of structures and systems have repeatedly been analysed through microscopy, including the parasite’s surface and tegumental structures, the attachment organs with a significant role in host-parasite interactions, its nervous and sensory system, the body’s musculature and mobility, along with its reproductive, excretory and alimentary systems [8,9]. The majority of these studies, however, were based on a single microscopic approach or were narrowly focused on a particular structure or system. Apparently, the investigation of morphological adaptations to parasitism in metazoan organisms requires a more complex approach using a combination of microscopy methods (e.g. [9,40]). Hence, the aim of this study was to provide a complex analysis of *P*. *homoion* adult-stage body architecture in relation to adaptation to an ectoparasitic life-style. Here, we describe those structures involved in parasite host-attachment, movement, host blood-sucking and excretion.

## Material and methods

### Material collection

Samples of *Paradiplozoon homoion* (Bychowsky et Nagibina, 1959) were collected from the gills of roach *Rutilus rutilus* (L.), bleak *Alburnus alb*urnus (L.) and gudgeon *Gobio gobio* (L.). The fish were caught by electrofishing or using gillnets in Mušov lowland reservoir (southern Moravia, Czech Republic) during the year 2013. The fish collection was carried out by external collaborators from Institute of Vertebrate Biology, Academy of Science, Czech Republic (wild fish collection of Institute of Vertebrate Biology is approved by certificate issued by Ministry of Agriculture No. 3OZ31162/2011-17214). Fish were transported in aerated original water to the laboratory facilities of Faculty of Science, Masaryk University, Brno, Czech Republic (Permit No. 16256/2015-MZE-17214). Fish were sacrificed by stunning and cutting the spine, and all efforts were made to minimize suffering (in accordance with the Act No. 246/1992 Coll., on Prevention of Cruelty to Animals). Gills were removed according to the standard protocol [41] and examined. This study was carried out in strict accordance with Act No.207/2004 of the Collections of Laws of the Czech Republic on the Protection, Breeding and Use of Experimental Animals. The study was approved by the Animal Care and Use Committee at the Faculty of Science, Masaryk University, Czech Republic and followed to Ministry of Education, Youth and Sports (Permit No. 13715/2011-30).

### Confocal laser scanning microscopy (CLSM)

Diplozoid worms were flat-fixed between microscopic slides in freshly prepared 4% paraformaldehyde in 0.1 phosphate buffered saline (PBS) for 4 h at 4°C and then transferred into fresh fixative. For the labelling of filamentous actin (F-actin), specimens were washed for 24 h in antibody diluent (AbD) containing 0.1 M PBS, 0.5% Triton X-100, 0.1% bovine serum albumin and 0.1% NaN_3_ at pH 7.4. The samples were subsequently incubated in phalloidin–tetramethylrhodamine B isothiocyanate (phalloidin-TRITC; Sigma-Aldrich, Czech Republic) and AbD (10μl/1ml) for 48 h at room temperature and then washed again in AbD for 24 h at 4°C. For double fluorescent labelling, specimens were fixed, washed and permeabilised for 48 h in 0.5% Triton X-100 (Sigma-Aldrich, Czech Republic). The samples were then incubated with mouse monoclonal anti-α-tubulin antibody (Clone B-5-1-2, Sigma-Aldrich, Czech Republic) at 4°C for six days, washed for 24 h in AbD and finally incubated with mouse polyvalent immunoglobulins (1:125) in PBS with 1% BSA at 37°C for four days. The specimens were then washed and incubated in TRITC-phalloidin as described above. Controls were labelled with FITC-conjugated secondary antibody only without the primary antibody. For localisation of cell nuclei, preparations were counterstained with either DAPI and mounted in 9:1 glycerol/PBS containing 2.5% 1,4-diazabicyclo [2.2.2] octane (DABCO, Sigma-Aldrich) or with Hoechst and mounted in VECTASHIELD^®^ (Vector Laboratories, USA). Gomori trichrome staining was used for 3D visualisation of sclerotised structures [42]. The hydrochloric carmine staining of whole-mount preparations follows published protocols [43].

All slides were examined and documented using an Olympus IX81 microscope equipped with a laser-scanning FluoView 500 confocal unit (Olympus FluoView 4.3 software) or an Olympus BX60 microscope with FluoView 1.26 (Fluoview 2.0 software). Some confocal micrographs were processed using Fiji software (an image-processing package based on ImageJ, developed at the National Institute of Health).

### Electron microscopy

For scanning electron microscopy (SEM), the specimens were first washed several times in tap water to remove any fish mucus, fixed in either hot 4% formaldehyde or 4% glutaraldehyde at 4°C for 24 h and postfixed for 1 h in 1% OsO_4_. The samples were subsequently dehydrated through a graded ethanol series and dried in a Pelco CPD II critical point drying apparatus (Bal-Tec) using liquid CO_2_. The dried samples were finally mounted on aluminium stubs with double-sided adhesive tape or disks, coated with gold in a Polaron E5100 sputter coating unit (Balzers) and examined in a MIRA 3 TESCAN SEM operating at 15 kV.

## Results

**Fig 1:**
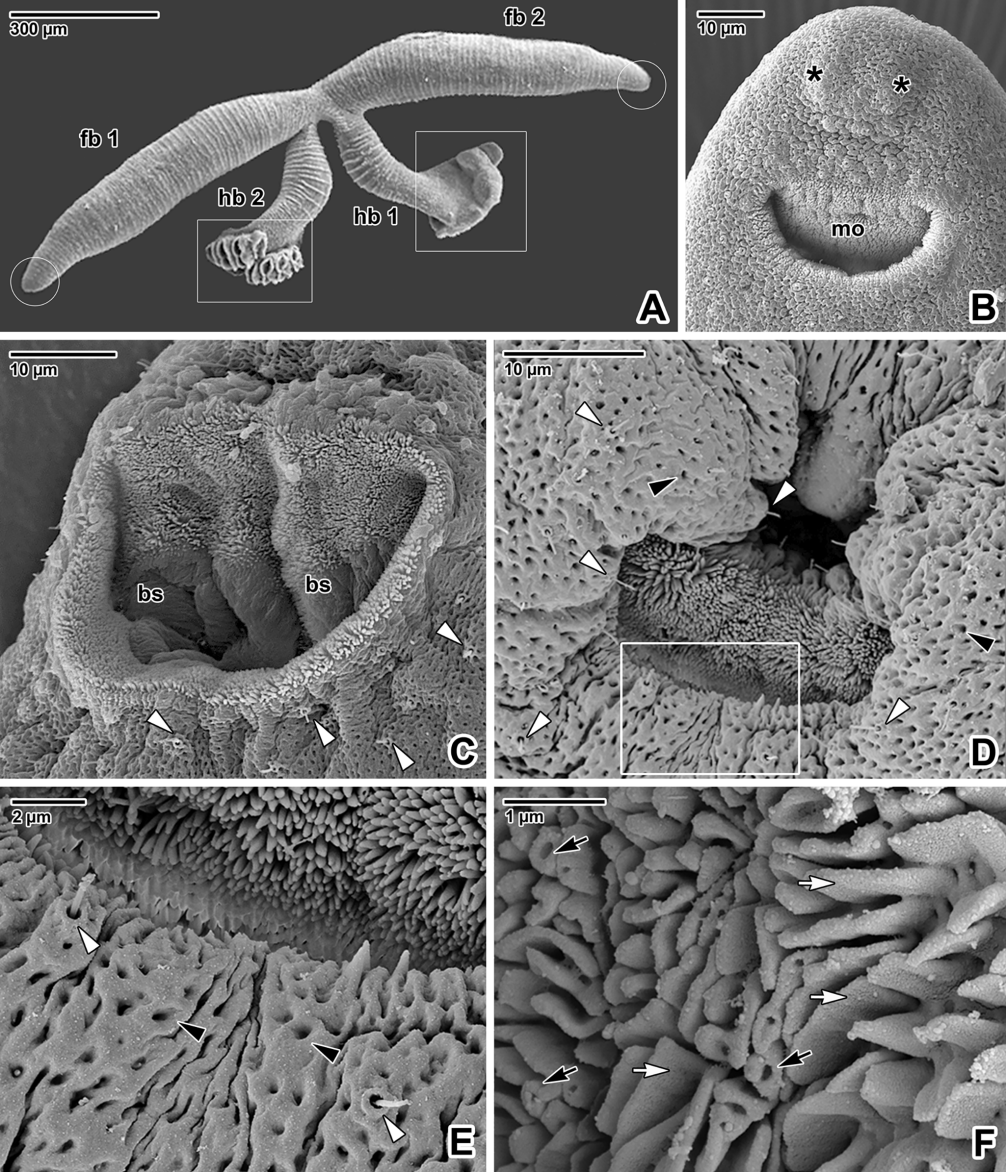
Surface topology of forebody tegumentary structures in *Paradiplozoon homoion* adults. **A)** Overall view of an adult. The white circles indicate the area of the mouths and the white rectangles the area of the haptors. SEM. **B)** Ventral view of the forebody, with a subterminal mouth and two apically located, round projections. SEM. **C)** Micrograph of an opened mouth revealing two buccal suckers and uniciliated sensory structures. SEM. **D)** The mouth area covered by numerous uniciliated sensory structures and pits. SEM. **E)** Detail of the mouth border (the area marked by the white rectangle in D). Note the tegument with numerous pits and sensory structures. SEM. **F)** Detail of the buccal cavity surface enlarged by numerous foliate and tubular digitations. SEM. *asterisks*–round projections, *black arrowheads*–pits, *black arrows*–tubular digitations, *bs*–buccal sucker, *fb 1*– forebody 1, *fb 2 –*forebody 2, *hb 1 –*hindbody 1, *hb 2 –*hindbody 2, *mo*–mouth, *white arrowheads*–uniciliated sensory structures, *white arrows*–foliate digitations.

This study focuses on individuals that have already paired and formed the juvenile/adult stages. As in other members of the family Diplozoidae, the body of the *P*. *homoion* in the adult stage typically resembles a letter X. This X-shaped body is comprised of two forebodies and two hindbodies along with the haptors of the two fused individuals (Fig 1A).

### Adult forebody

The mouth is situated subterminally on the ventral side of the forebody, with two rounded projections protruding above it (Fig 1B). The surface of the tegument around the mouth bears numerous uniciliated sensory structures and pits (Fig 1C–1E). The buccal cavity is equipped with two well-developed buccal suckers (Fig 1C). The mouth is limited by a brush border (Fig 1C). The surface of the buccal cavity is substantially enlarged by abundant foliate and tubular digitations (Fig 1F).

**Fig 2:**
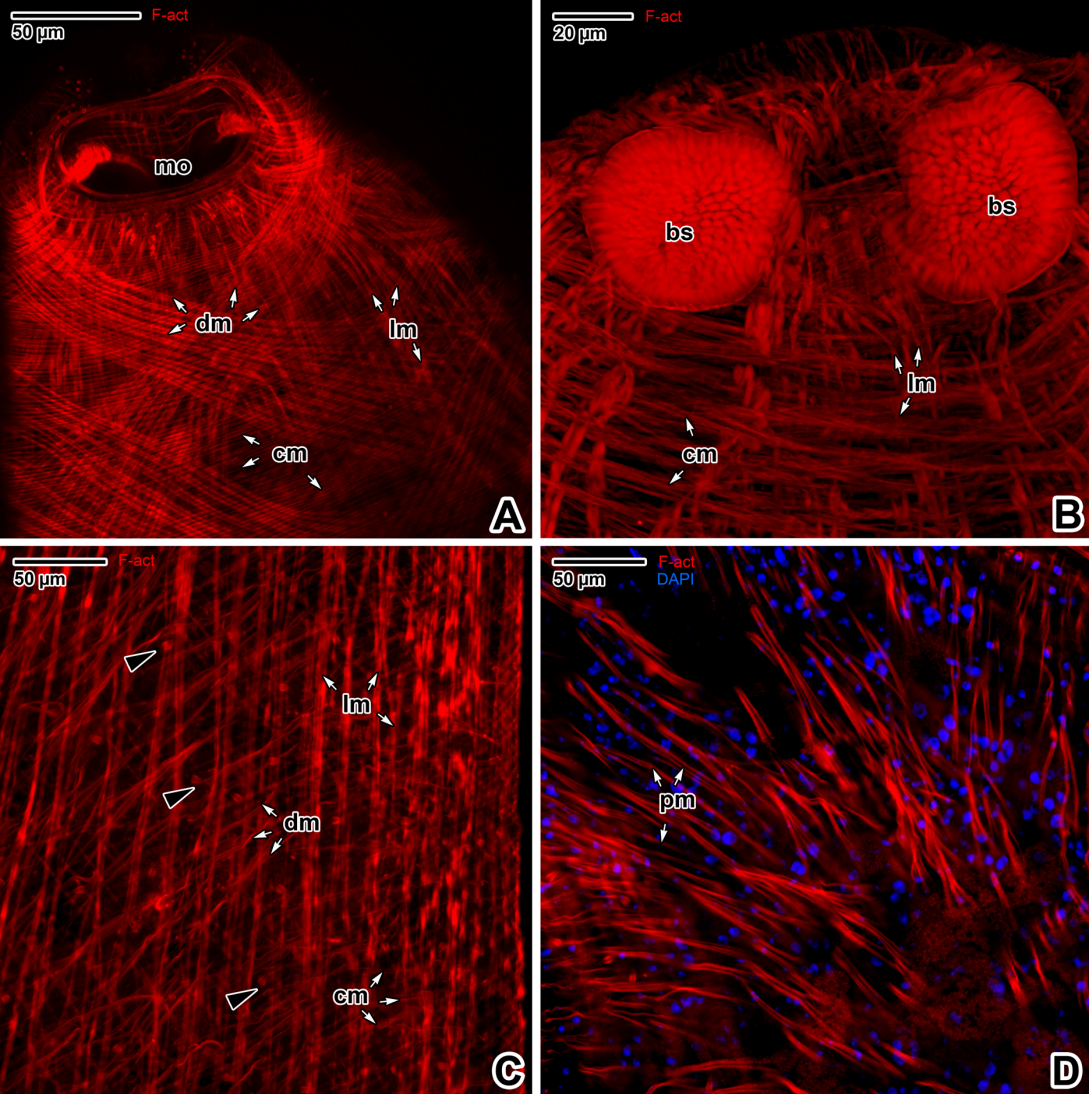
Forebody wall musculature of *Paradiplozoon homoion* adults. **A-B)** General views of the body wall musculature arrangement. CLSM, phalloidin-TRITC. **C-D)** Detail of the body wall musculature. CLSM, phalloidin-TRITC (C) and phalloidin-TRITC/DAPI (D). A-B are composite views created by flattening a series of optical sections, while C-D represent single optical sections. *black arrowheads*–flame cells, *bs*–buccal suckers, *cm*–circular muscles, *dm*–diagonal muscles, *lm*–longitudinal muscles, *mo*–mouth opening, *pm*–perpendicular muscles.

**Fig 3:**
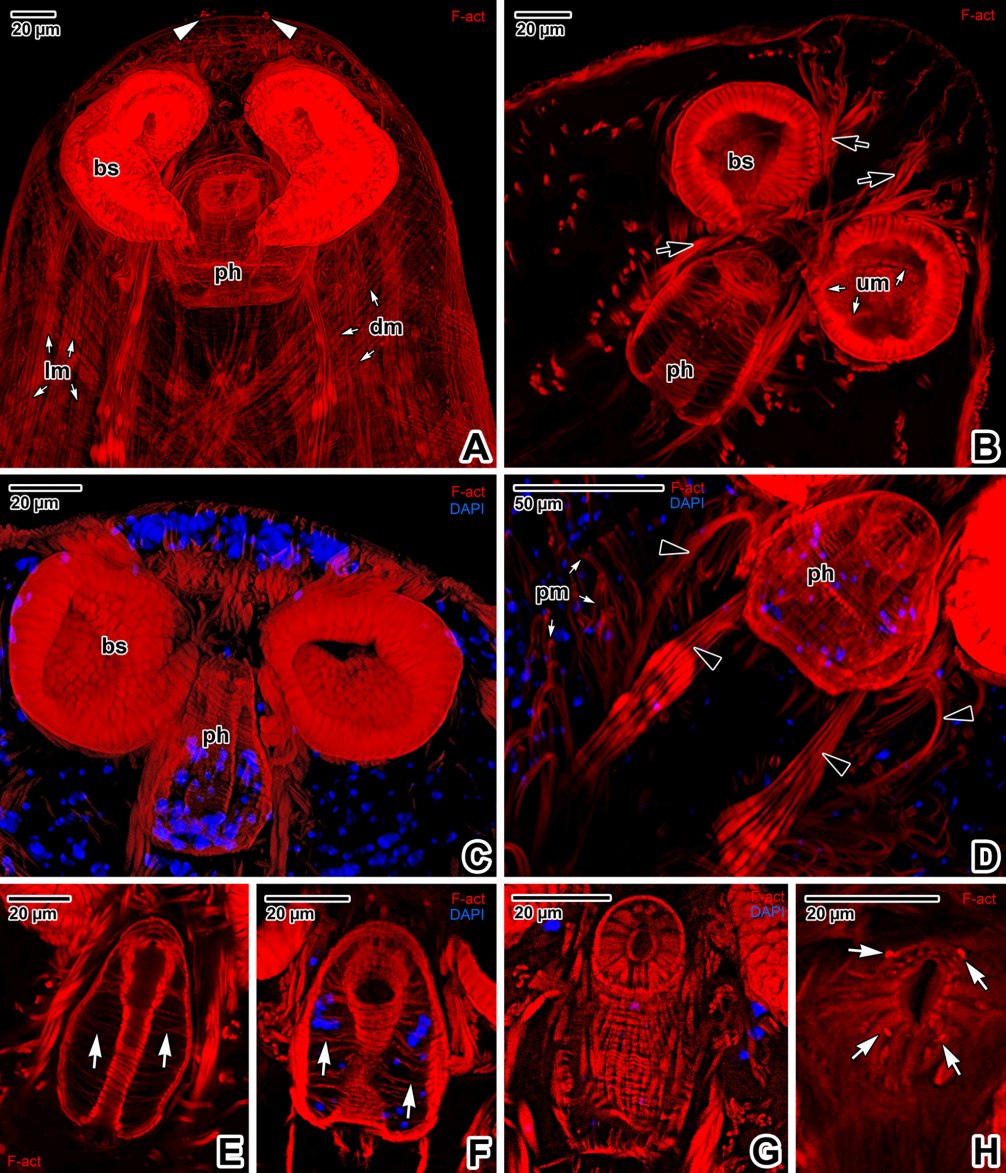
The main muscular organs in the forebody of *Paradiplozoon homoion* adults. **A)** General view of the forebody. CLSM, phalloidin-TRITC. **B-C)** The forebody in median plane optical sectioning. Note the muscles forming thin trabeculae in the forebody apical part and the numerous cells with prominent nuclei located between the trabeculae (C). CLSM, phalloidin-TRITC (B) and phalloidin-TRITC/DAPI (C). **D)** Detail showing the pharynx muscle wall arrangement and the muscles controlling pharynx movement. CLSM, phalloidin-TRITC/DAPI. **E-F)** Various optical sections of the pharynx showing the transverse trabeculae and pharyngeal cell nuclei. **G-H)** Apical view of the pharynx. CLSM, Phalloidin-TRITC (E, H) and phalloidin-TRITC/DAPI (F, G). **A-H** are composite views created by flattening a series of optical sections. *black arrowheads*–muscles controlling the pharynx, *black arrows*–muscles controlling the buccal suckers, *bs*–buccal suckers, *dm*–diagonal muscles of the body wall, *lm*–longitudinal muscles of the body wall, *ph*–pharynx, *pm*–muscle fibres fixed perpendicularly to the tegument, *um*–U-shaped muscle bundle of a sucker, *white arrowheads*–two sensory structures localised in the area of the round projections, *white arrows* in E, F–trabeculae, *white arrows* in H–four round structures.

Fluorescent labelling of F-actin with phalloidin allowed visualisation of the forebody muscular layer arrangement (Fig 2A–2D), revealing muscular organs such as buccal suckers and pharynx (Figs 2B and 3A–3H). The forebody wall musculature is arranged in three layers; an external circular muscle layer, a deeply situated longitudinal layer and several diagonal layers (Figs 2A–2C and 3A). In addition, numerous perpendicular, long and thin muscles interconnect the tegument and parenchyma (Fig 2D). The apex of the forebody is segmented by muscular trabeculae and filled with numerous prominent nuclei (Fig 3B and 3C).

The most prominent structures in the apical part of the forebody were the two oval buccal suckers (Fig 3A–3C). The border of the bowl-like buccal sucker is clearly demarcated by densely arranged radial muscle fibres, while a transverse U-shaped muscle bundle is visible at its centre (Fig 3B). The suckers are controlled by muscles connected to the body wall musculature and individual muscle fascicles oriented towards the forebody apex or the pharynx, with some localised obliquely between the two suckers (Fig 3B). The pharynx is a conspicuous elongated muscular organ situated in the medial plane near to the posterior margins of the buccal suckers. The pharynx is composed of lumen passing through its central part and a muscular wall, with massive muscle bundles enabling movement during blood sucking (Fig 3A–3H). The circular musculature of the pharyngeal lumen is fixed to the pharynx wall by numerous trabeculae (Fig 3E and 3F). Numerous cell nuclei located between the radial muscular trabeculae appear to be those of glandular pharyngeal cells (Fig 3C and 3F). The pharynx wall musculature is arranged into layers of radial, longitudinal and circular fibres (Fig 3A–3G). The anterior part of the pharynx is lined with an obvious muscular collar bearing four F-actin-rich oval structures (Fig 3H).

**Fig 4:**
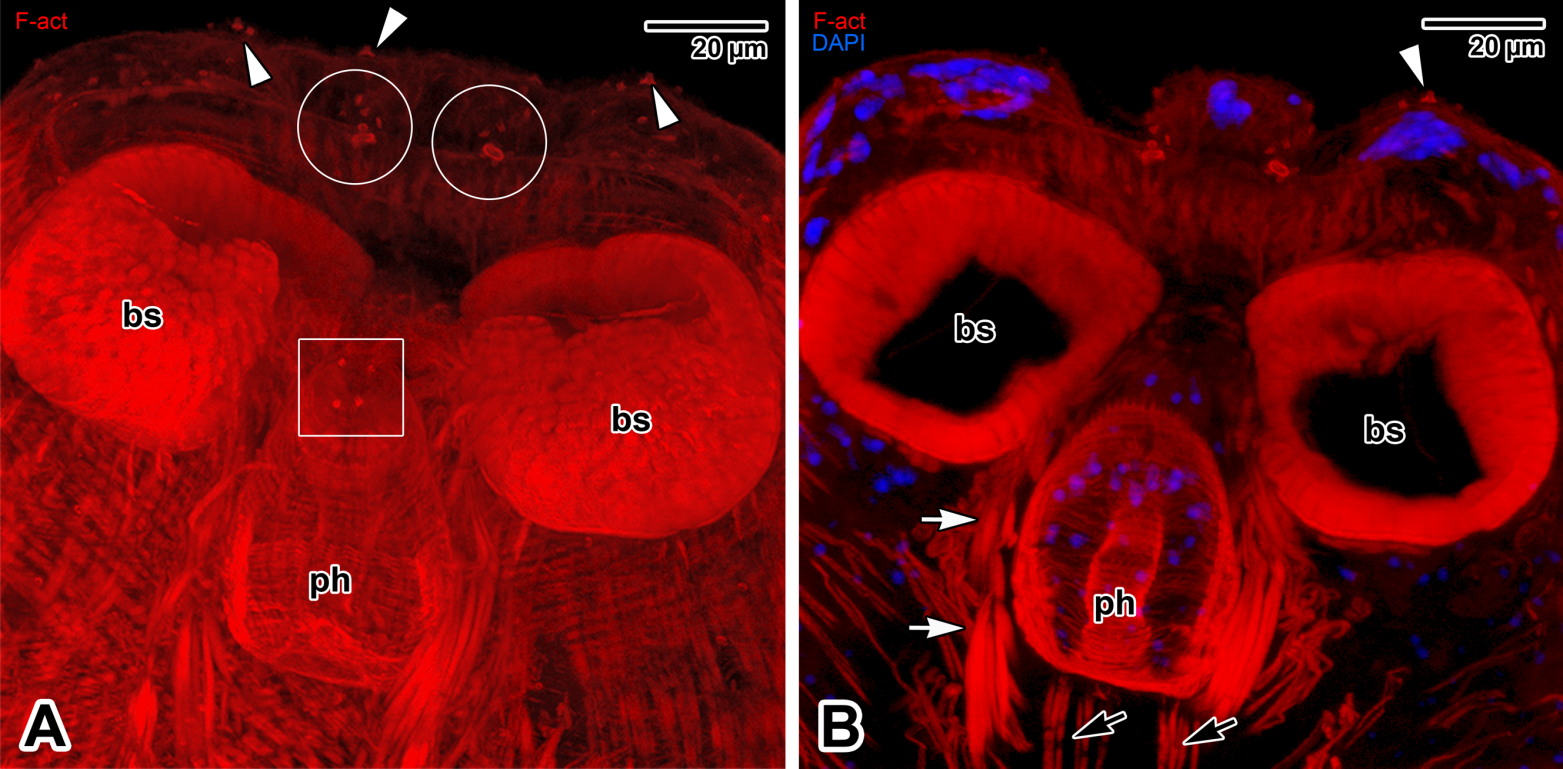
Forebody of *Paradiplozoon homoion* adults, with emphasis on the apical sensory and circular structures. **A)** The apical part of the forebody, revealing the distribution of sensory structures and a group of circular structures (*encircled*). The white rectangle indicates the area of the apical part of the pharynx containing four round structures. CLSM, phalloidin-TRIC. The micrograph is a composite view created by flattening a series of optical sections. **B)** Single medial plane optical section of the forebody. CLSM, phalloidin-TRIC/DAPI. *black arrows*–muscles controlling the pharynx, *bs–*buccal sucker, *ph*–pharynx with four circular openings, *white arrowheads*–sensory structures, *white arrows*–muscles controlling the buccal suckers.

**Fig 5:**
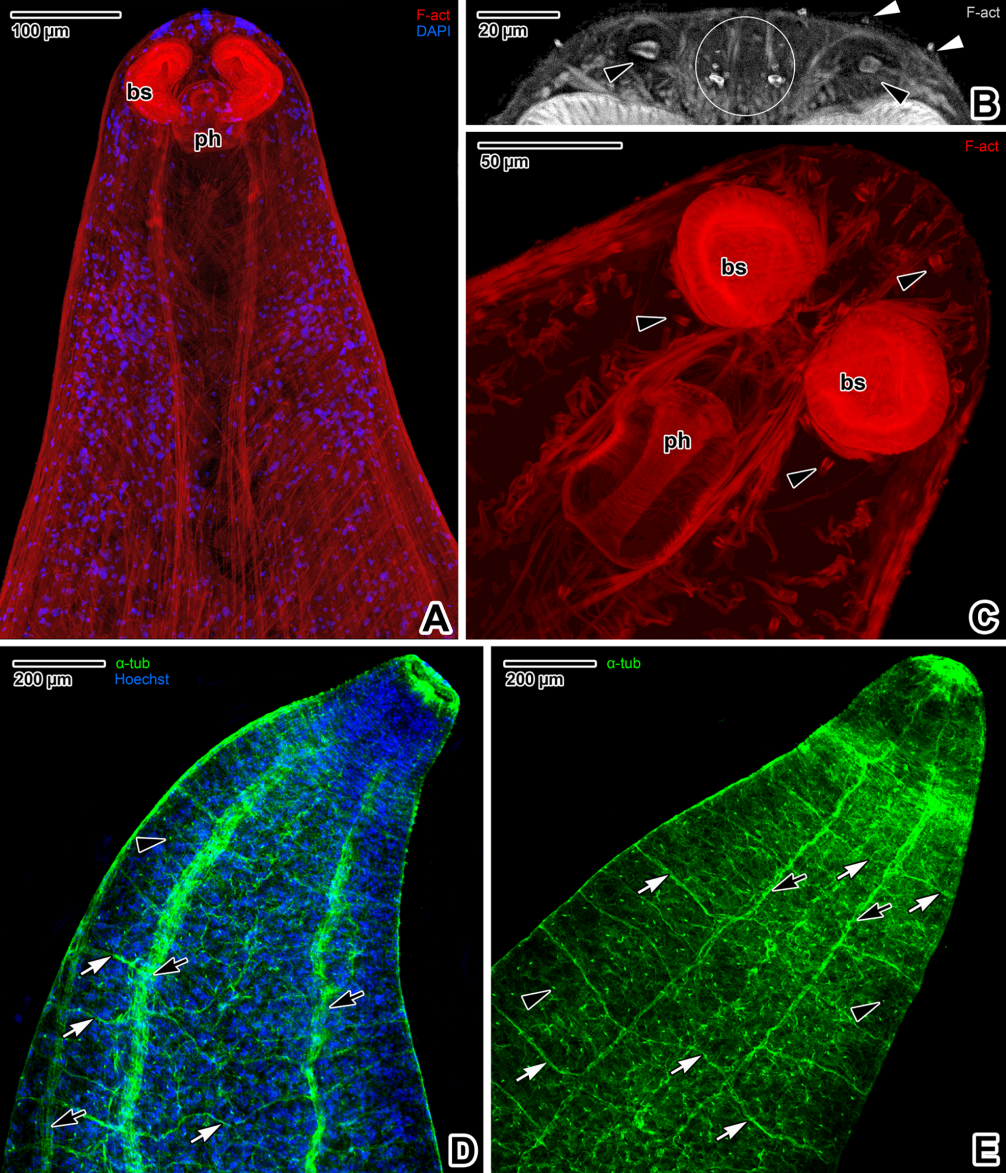
Forebody of *Paradiplozoon homoion* adults, with emphasis on the muscular, excretory and nervous systems. **A)** Total view of the musculature and the main muscular organs of the forebody. CLSM, phalloidin-TRITC/DAPI. **B)** Detail of two flame cells located above the buccal suckers and the area with apical circular structures (*encircled*). CLSM, phalloidin-TRITC (the output image is uncoloured). **C)** Distribution of flame cells in the area around the pharynx and buccal suckers. CLSM, phalloidin-TRITC. **D-E)** Forebody nerve cords and excretory system. CLSM, IFA-FITC/Hoechst (D) and IFA-FITC (E). **A, D** and **E** are composite views created by flattening a series of optical sections, while **B** and **C** represent single median optical sections. *bc*–buccal sucker, *black arrowheads*–flame cells, *black arrows*–longitudinal (dorsal and ventral) nerve cords, *ph*–pharynx, *white arrowheads*–uniciliated sensory structures, *white arrows*–transverse connective cords.

Phalloidin staining enabled detection of two groups of circular structures (of unknown origin and function) symmetrically arranged in the middle of the forebody apical end (Figs 4A, 4B and 5A), sensory structures (Figs 4A, 4B and 5B) and flame cells, these representing the basic elements of an excretory system (Fig 5B and 5C).

**Fig 6:**
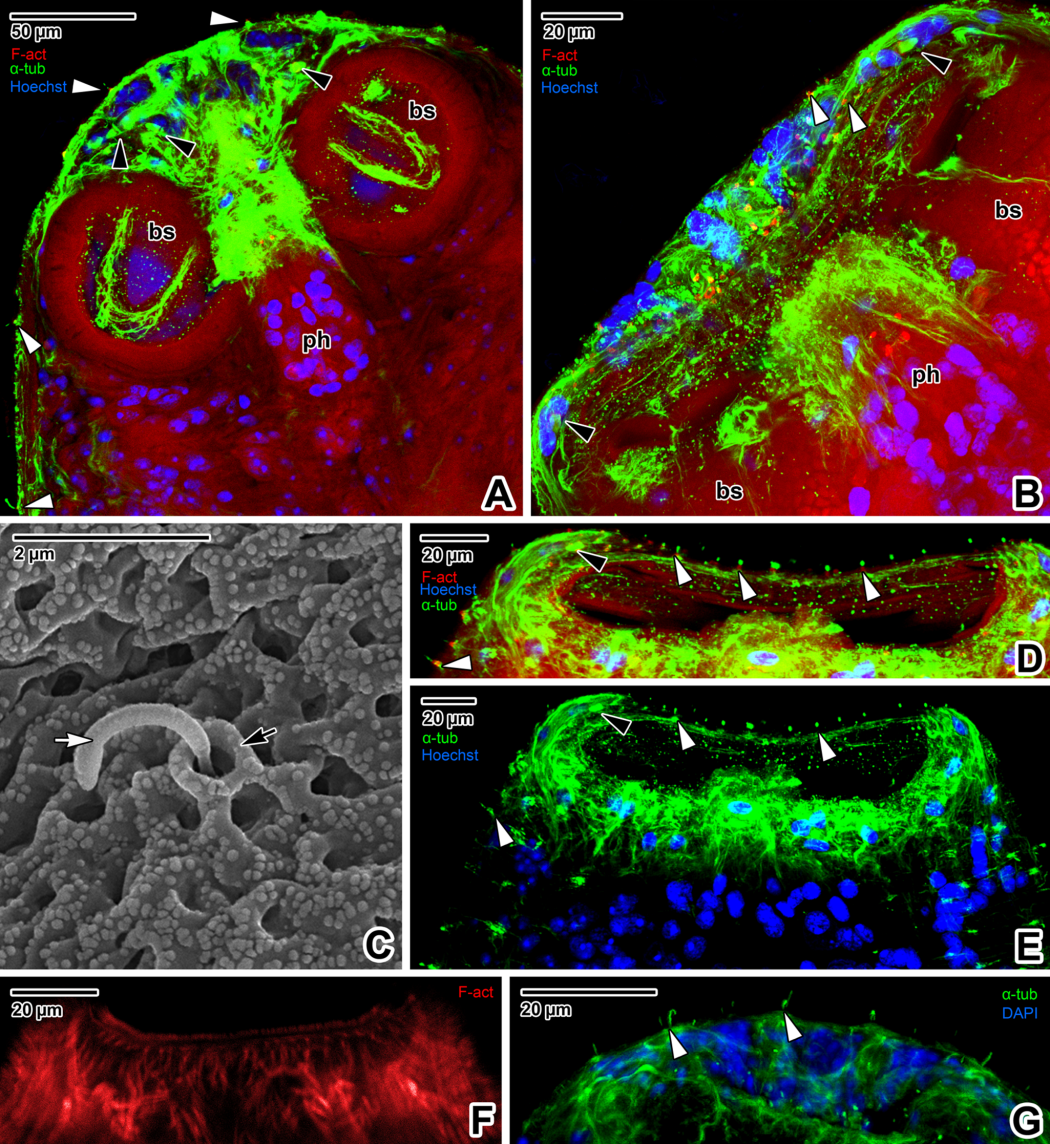
Mouth border of *Paradiplozoon homoion* adults. **A)** Median plane optical sectioning of the forebody. Note the accumulation of α-tubulin associated with the forebody apical part and buccal suckers. CLSM, IFA-FITC/phalloidin-TRITC/Hoechst. **B)** View of the forebody showing the tubulin-rich apical end. CLSM, IFA-FITC/phalloidin-TRITC/Hoechst. **C)** Detail of a uniciliated sensory structure with a raised circular rim and one long cilium. SEM. **D-E)** A different optical section of the specimen in B) revealing the tubulin-rich border of the mouth opening and the distribution of uniciliated sensory structures. CLSM, IFA-FITC/phalloidin-TRITC/Hoechst (D) and IFA-FITC/Hoechst (E). **F)** Arrangement of the muscle fibres around the border of the mouth opening. CLSM, phalloidin-TRITC. **G)** Detail of uniciliated sensory structures in the forebody apical end. CLSM, IFA-FITC/DAPI. **A-B, D-E** and **G** are composite views created by flattening a series of optical sections, while **F** represents a single optical section. *black arrow*–raised circular rim, *black arrowheads*–flame cells, *bs*–buccal suckers, *ph*–pharynx, *white arrow*–cilium, *white arrowheads*–uniciliated sensory structure.

**Fig 7:**
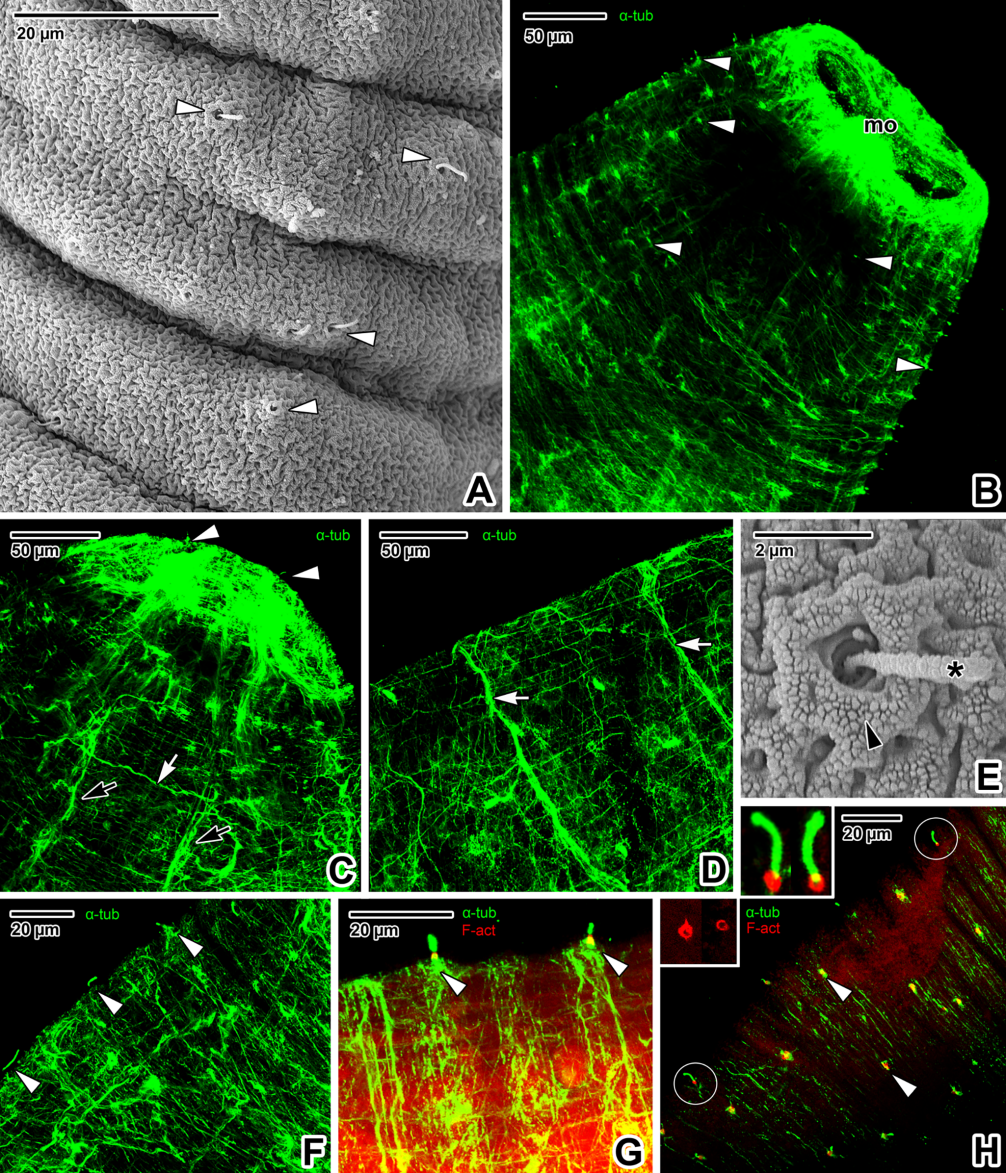
Forebody innervation in *Paradiplozoon homoion* adults. **A)** Tegumentary ridges and uniciliated sensory structures. SEM. **B)** Micrograph revealing the distribution of uniciliated sensory structures. CLSM, IFA-FITC. **C-D)** Longitudinal and transverse connective nerve cords. CLSM, IFA-FITC. **E)** Detail of the sensory structure, with visible cilium anchoring. SEM. **F-G)** Arrangement of uniciliated sensory structures and peripheral nerve fibres within the tegumentary ridges. CLSM, IFA-FITC (F) and IFA-FITC/phalloidin-TRITC (G). **H)** Superficial distribution of uniciliated structures, a view comparable with B). The inset to the right shows a detail of two sensory structures from H) (*encircled*), while the left inset shows the circular rim rich in F-actin only; both views are magnified five times. CLSM, IFA-FITC/phalloidin-TRITC. **A**, **C**, **D**, **F** and **G** are composite views created by flattening a series of optical sections, while **H** represents a single median optical section. *asterisk*–cilium, *black arrowhead*–circular rim, *black arrows*—longitudinal nerve cords, *mo–*mouth opening, *white arrowheads*–uniciliated sensory structures, *white arrows*–transverse connective cords.

Immunofluorescent labelling of α-tubulin localised the forebody nerve cords, and especially the longitudinal dorsal and ventral cords and transverse connective cords (Fig 5D and 5E). The α-tubulin antibody also enabled visualisation of the excretory system, comprising a number of flame cells distributed between the transverse nerve connectives in the apical part (Fig 5D and 5E). The apical part of the forebody, including the U-shaped middle part of buccal suckers (Fig 6A), appears to be tubulin-rich (Figs 6A, 6B, 6D, 6E, 6G, 7B and 7C). Several preparations revealed an ability of parasite to retract the pharynx and buccal suckers into the body, resulting in a half-closed appearance of the mouth (Figs 6B, 6D–6G and 7B). Concentration of α-tubulin around the mouth border confirms rich innervation of this region (Fig 6D, 6E and 6G), with regularly arranged muscle fibres anchored to the peripheral rim (Fig 6F). Numerous, regularly distributed uniciliated sensory structures with a raised circular rim and one long cilium were detected in the mouth area (Fig 6C). The circular rim is formed of F-actin (Fig 6B and 6D), while the cilium is strongly labelled for α-tubulin (Fig 6D, 6E and 6G).

The forebody surface is obviously folded, creating transverse ridges with shallow pits (Fig 7A). The tegument covering the forebody bears numerous uniciliated sensory structures distributed along the transverse ridges at regular intervals (Fig 7A, 7B and 7E–7H). Immunofluorescent labelling of α-tubulin indicated that innervation of the forebody comprises longitudinal nerve cords interconnected with transverse cords (Fig 7C and 7D). Peripheral innervation of the forebody forms a dense mesh of fine nerve fibres surrounding the main nerve cords (Fig 7B–7D). The peripheral nerve fibres are associated with tegumentary ridges with individual uniciliated sensory structures (Fig 7B, 7F and 7G). The arrangement of the single uniciliated receptor is similar to the sensory structures localised around the mouth. The circular rim is formed of F-actin (Fig 7G and 7H) and the tubulin-rich cilium is anchored by radially organised septa embedded in the tegument (Fig 7E).

**Fig 8:**
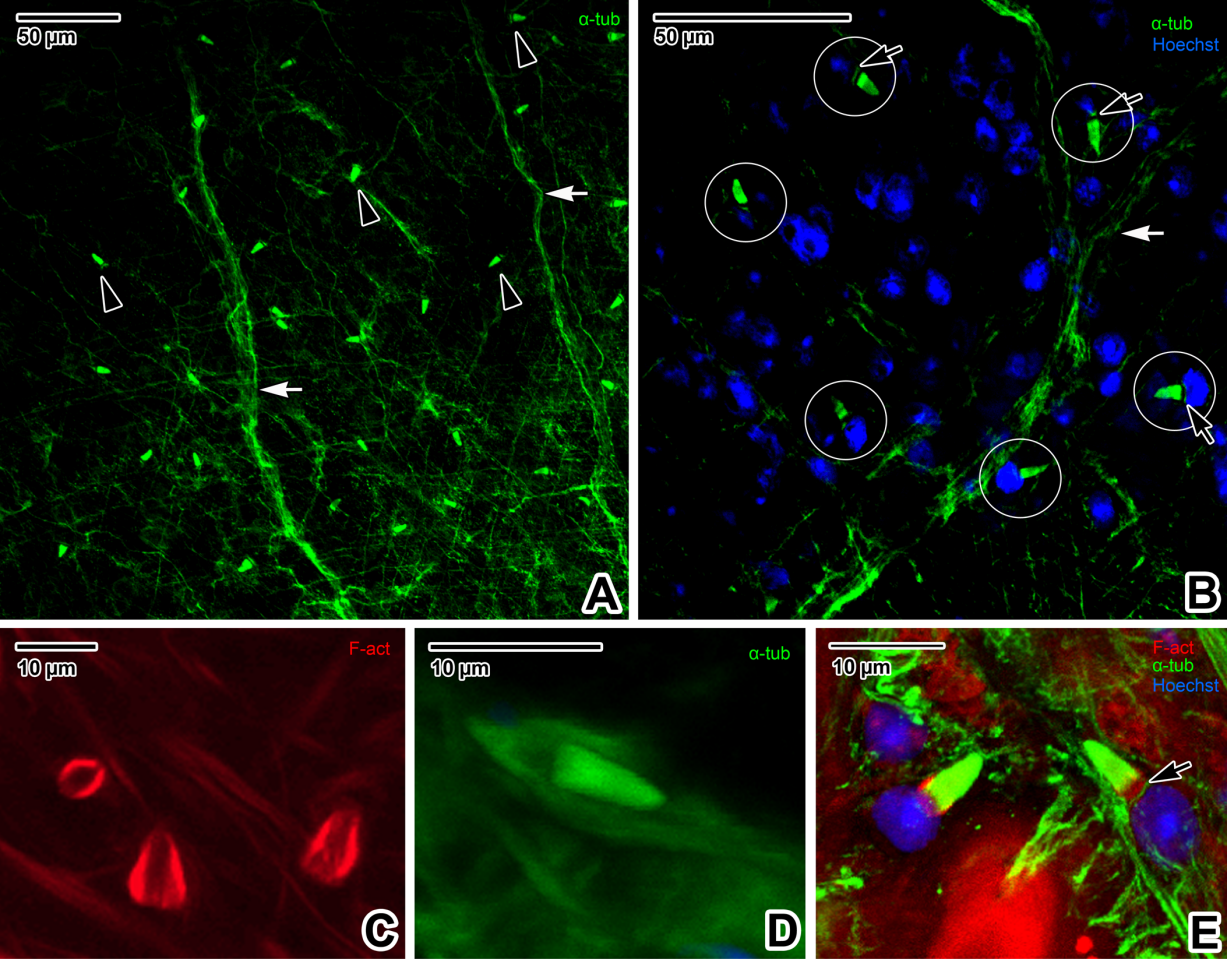
Visualisation of the excretory system of *Paradiplozoon homoion* adults using α-tubulin immunolabeling. **A)** Micrograph showing the distribution of flame cells and peripheral nerve fibres in the forebody region. CLSM, IFA-FITC. **B)** Flame cells (e*ncircled*) counterstained with Hoechst to indicate the nuclei of terminal cells. Note the green stained ciliated tufts and rootlets. CLSM, IFA-FITC/Hoechst. **C)** Detail of the flame cells. The barrel non-ciliated part involves both the terminal and adjacent canal cell. CLSM, phalloidin-TRITC. **D)** Detail of one flame cell with the ciliated tuft of the terminal cell. CLSM, IFA-FITC. **E)** Detail of two flame cells. CLSM, IFA-FITC/phalloidin-TRITC/Hoechst. **A** and **B** are single median optical sections, while **C** and **D** are composite views created by flattening a series of optical sections. *black arrowheads*–flame cells, *black arrows*–roots of tuft cilia, *white arrows*–transverse connective cords.

In Platyhelminthes, the protonephridial excretory system generally consists of terminal organs, i.e. flame cells consisting of terminal cells and adjacent canal cells, and a system of interconnected collecting ducts opening to the body surface [44]. However, immunofluorescent labelling of α-tubulin was only able to reliably visualise abundant flame cells, which are regularly distributed along the entire forebody (Fig 8A, 8B, 8D and 8E). The prominent flame cell nucleus was easily detected through Hoechst counterstaining (Fig 8B and 8E). The ciliated tuft (i.e. flame) and roots of the cilia were conspicuously labelled by the α-tubulin antibody (Fig 8B, 8D and 8E). Labelling of F-actin revealed the non-ciliated, barrel-shaped part of the flame cell, consisting of both terminal and adjacent canal cells (Fig 8C and 8E).

### Adult hindbody

**Fig 9:**
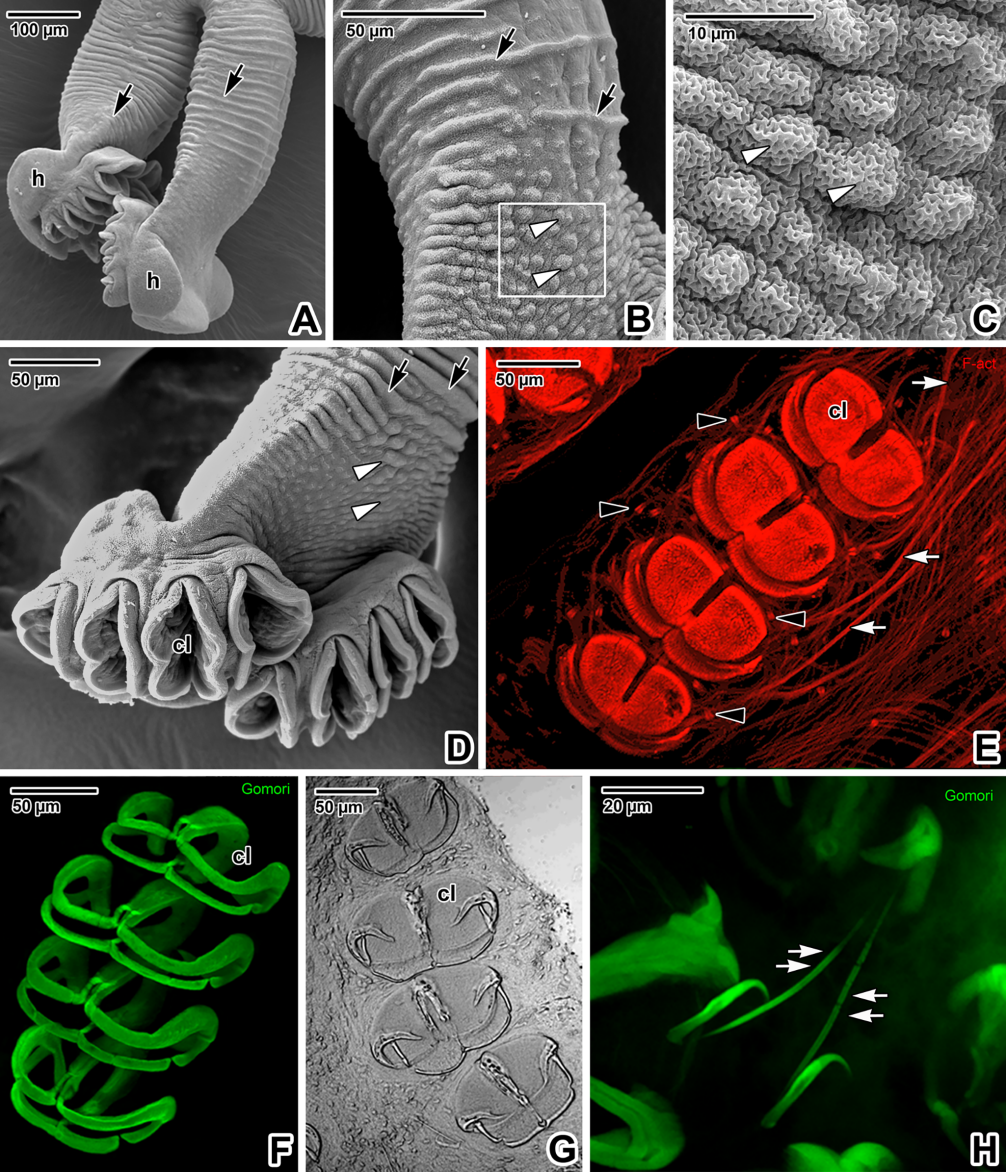
Hindbody of *Paradiplozoon homoion* adults. **A)** Lateral view of both hindbodies with prominent haptors. SEM. **B)** Detail of tegumentary papillae and the folds covering the middle part of the hindbody. SEM. **C)** Detail of the tegument with papillae. The micrograph shows the area marked by a white rectangle in B). SEM. **D)** The haptor, with eight clamps organised in two rows. SEM. **E)** Musculature of four clamps with operating muscle bundles. Note the distribution of flame cells. CLSM, phalloidin-TRITC. **F)** 3D visualisation of the clamp sclerites. CLSM, Gomori staining. **G)** Clamp sclerites. LM, bright field. **H)** Pair of marginal hooks localised between the two rows of clamps. CLSM, Gomori staining. **D**, **E**, **F** and **H** represent composite views created by flattening a series of optical sections. *black arrowheads*–flame cells, *black arrows*–tegumentary folds, *cl*–clamps, *double white arrows*–marginal hooks, *h*–haptor, *white arrowheads*–tegumentary papillae, *white arrows–*extrinsic muscle bundles.

**Fig 10:**
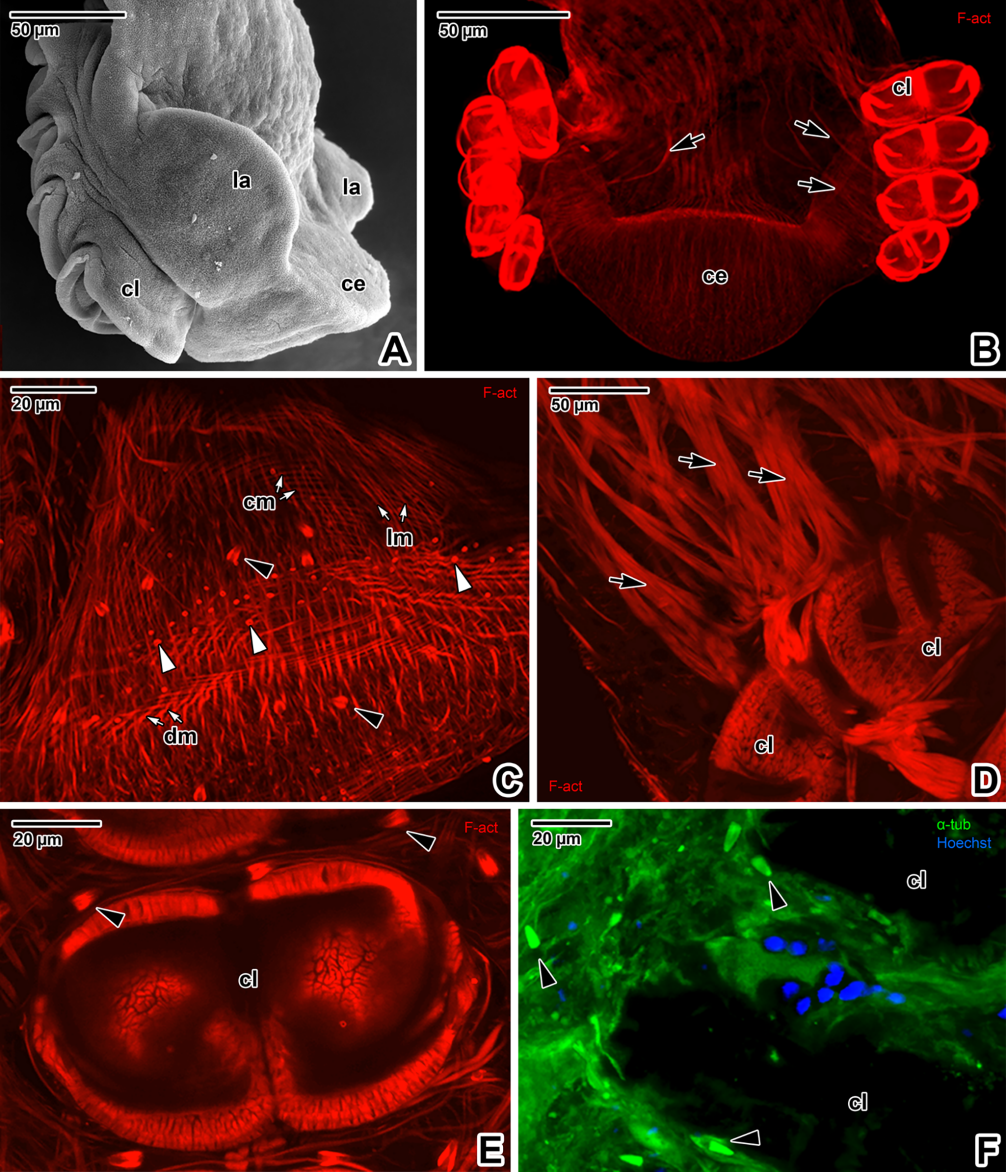
Hindbody with haptor in *Paradiplozoon homoion* adults, with emphasis on musculature. **A)** Lateral view of the haptor, with one row of four clamps and three lobed structures (one central and two lateral lobes). SEM. **B)** Muscular haptor equipped with four pairs of clamps with extrinsic muscle bundles and a central lobe. CLSM, phalloidin-TRITC. **C)** Central lobe of the haptor, with numerous flame cells. Note the muscle arrangement (longitudinal, circular and diagonal). CLSM, phalloidin-TRITC. **D)** Detail of the massive muscle bundles controlling the clamp. CLSM, phalloidin-TRITC. **E)** Detail of the clamp musculature surrounded by flame cells. Note the strong F-actin labelling localised in the barrel part of the flame cells. CLSM, phalloidin-TRITC. **F)** α-tubulin labelling of flame cell ciliated tufts located near the clamps. CLSM, IFA-FITC/DAPI. **B**-**F** represent composite views created by flattening a series of optical sections. *black arrows*–extrinsic muscle bundles, *black arrowheads*–flame cells, *ce–*central lobe, *cl*–clamps, *cm*–circular muscles, *dm*—diagonal muscles, *la–*lateral lobes, *lm*–longitudinal muscles, *white arrowheads*–sensory structures.

**Fig 11:**
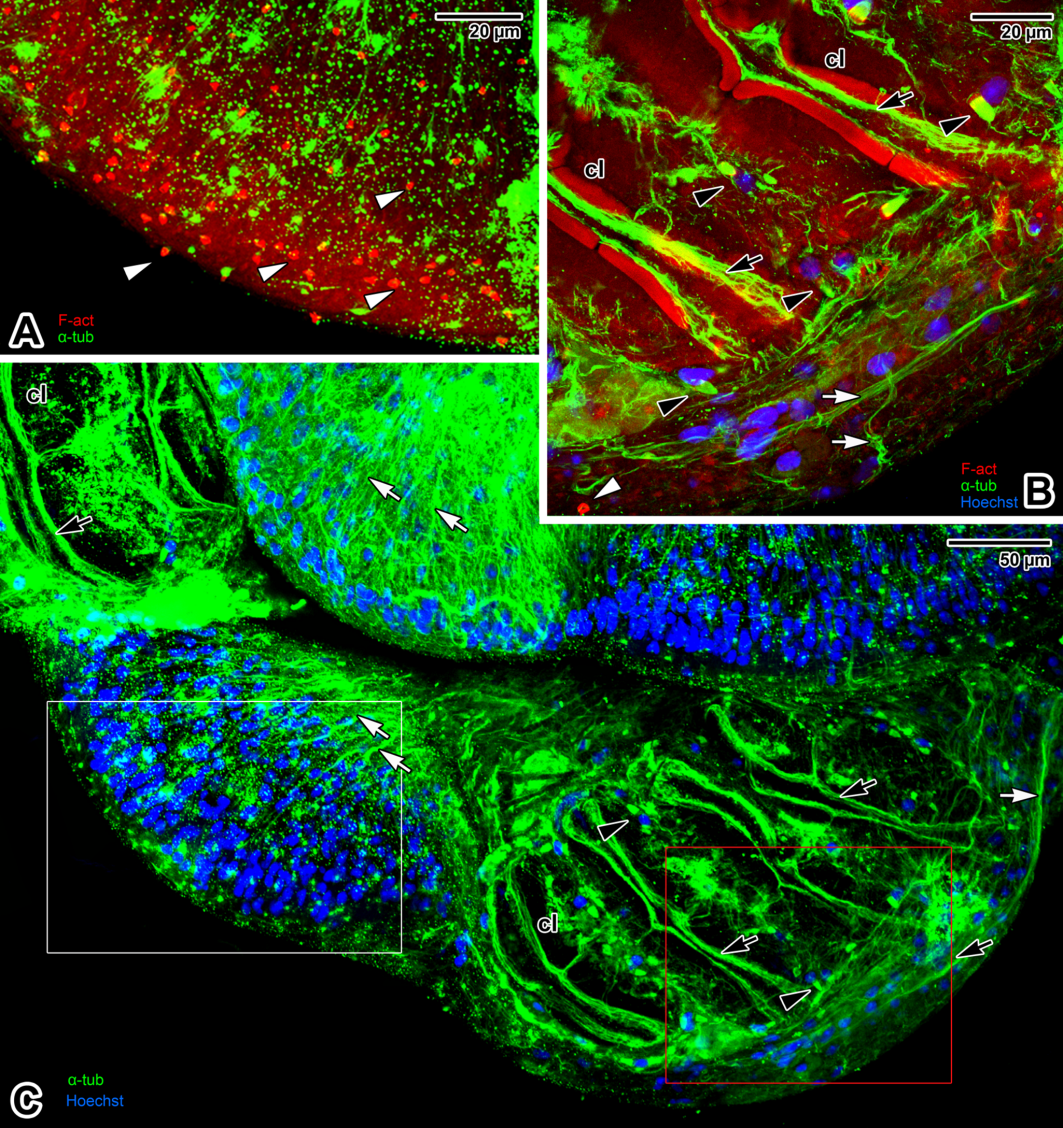
Hindbody with haptor in *Paradiplozoon homoion* adults, with emphasis on innervation. **A)** Detail of the haptor central lobe. Note the distribution of uniciliated sensory structures (F-actin) and peripheral nerve fibre endings (α-tubulin). The micrograph shows the area marked by a white rectangle in C). CLSM, IFA-FITC/phalloidin-TRITC. **B)** Double F-actin and α-tubulin labelling of the region surrounding the two clamps. The micrograph shows the area marked by a red rectangle in C). The dense red structures represent autofluorescence of the clamp sclerites. CLSM, IFA-FITC/phalloidin-TRITC/Hoechst. **C)** General view of the hindbody labelled for α-tubulin and counterstained with Hoechst. CLSM, IFA-FITC/Hoechst. **A**-**C** represent composite views created by flattening a series of optical sections. *black arrowheads*–flame cells, *black arrows*–innervation of clamps, *cl*–clamps, *white arrowheads*–uniciliated sensory structures, *white arrows*–peripheral nerve fibres.

The major part of the hindbody surface is covered with transverse, discontinuous tegumentary folds (Fig 9A, 9B and 9D), while papilla-like structures prevail in the middle part of the ventral side above the haptor (Fig 9B–9D). The haptor bears four pairs of clamps organised in two rows on its ventral side (Fig 9D). These clamps are covered with a thin layer of tegument (Figs 9D and 10A). The base of the clamps is comprised of both musculature and sclerotised parts. The clamp sclerites form a pincer-like mechanism allowing for opening and closing movements (Fig 9F and 9G). The clamp musculature, controlled by well-developed muscle bundles, enables the sclerites to move and attach the clamps to host tissue (Figs 9E, 10B and 10D). Gomori staining enabled 3D visualisation of both the clamp sclerites and the pair of marginal hooks between the rows of clamps (Fig 9F and 9H). The dorsal side of the haptor is divided into three (one central and two lateral) lobed structures (Fig 10A). The haptor’s musculature appears massive (especially in the central lobe) and is arranged as longitudinal, circular and diagonal muscle fibres (Fig 10B and 10C). The flame cells are more abundant in the central lobe and surrounding the clamps (Figs 10C, 10E, 10F and 11B). The sensory structures, distinguishable due to an F-actin-rich rim, are also frequent in the central lobe (Fig 10C). Situated near the haptor, the nervous system (strongly labelled for α-tubulin) is represented by peripheral nerve fibres with a mesh-like arrangement in the central area that most likely innerve the abundant sensory structures on the tegument surface (Fig 11A and 11C). The clamps are well innervated and strong bundles of nerve fibres surround and copy the sclerites of the clamp jaws (Figs 11B and 10C). The middle part, above the haptor, is packed with numerous cells and is densely innervated (Fig 11C).

### Cellular morphology of adults stained with hydrochloric carmine

**Fig 12:**
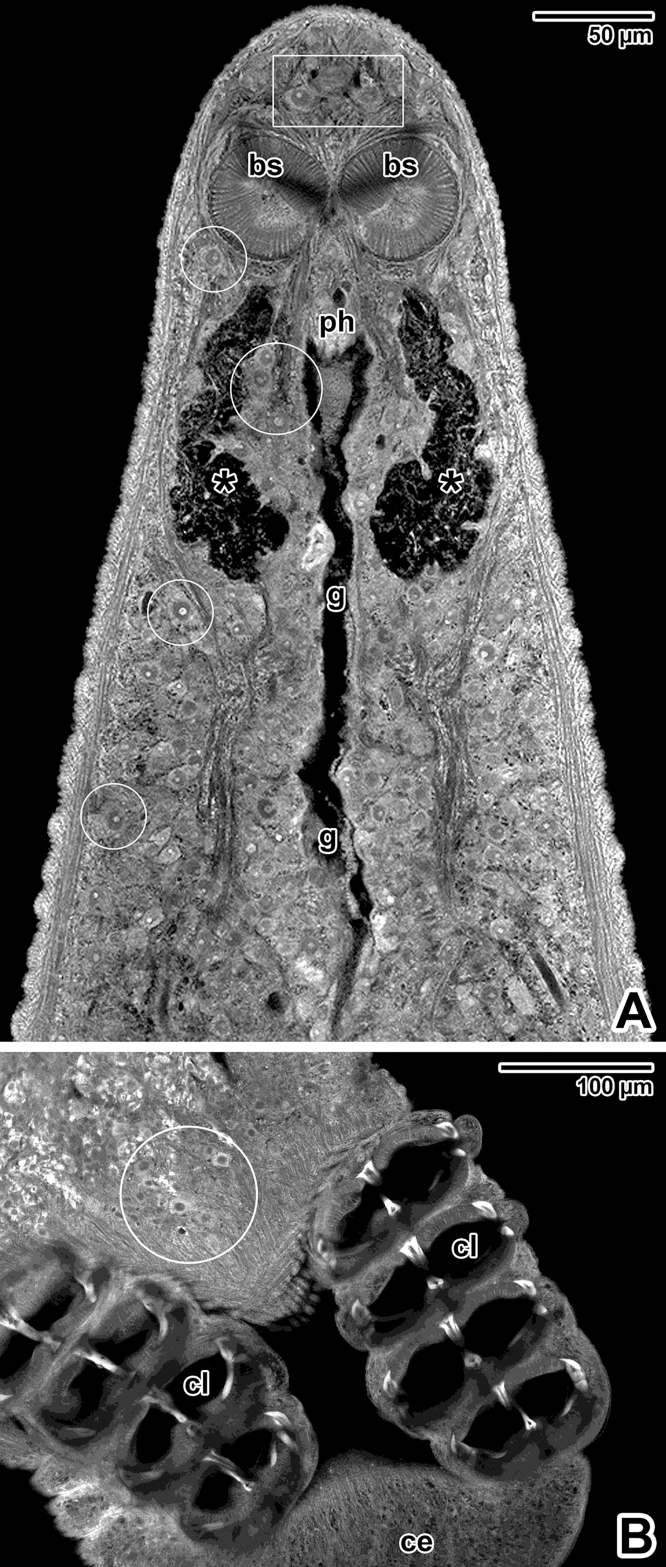
Cellular morphology of *Paradiplozoon homoion* adults stained with hydrochloric carmine. **A)** Micrograph showing the forebody region. The white circles indicate some of the putative unicellular glands. The white rectangle demarks the putative glands in the area of apical circular structures. CLSM, output image not coloured. **B)** Micrograph showing the haptor. The accumulation of putative gland cells is demarcated by white circle. CLSM, output image not coloured. A-B are composite views created by flattening a series of optical sections. *asterisks*–paired club-shaped sacs, *bs*–buccal suckers, *ce–*central lobe, *cl*–clamps, *g*–foregut, *ph*–pharynx.

This staining revealed the presence of large gland-like cells with prominent nuclei. Majority of these cells are randomly scattered over the body, with increased occurrence in the forebody and haptor regions (Fig 12A and 12B). Of special interest is the accumulation of putative gland cells located in the area of apical circular structures (described above), and around the pharynx. Furthermore, we detected a pair of club-shaped sacs lying laterally to the pharynx and opening towards the prepharyngeal/pharyngeal region. These so far undetected structures exhibit no staining affinity to carmine and appear dark without fluorescence signal, indicating the absence of cell structures.

## Discussion

Ectoparasitic diplozoid monogeneans exhibit a range of unique biological characteristics and sophisticated functional adaptations to their bloodsucking life style. The most significant of these structures are those related to host searching, attachment, feeding/metabolism, pairing and protection against host responses [12,17]. As previous microscopy studies have shown the benefits of CLSM analysis with fluorescent labelling for detection of specific structures, we used phalloidin labelling of F-actin for visualisation of muscle structures and tubulin staining for detection of the nervous or excretory systems [40,45].

The tegument is the primary surface for host-parasite interaction during the search for a suitable niche on the host’s gills and also plays an essential role during contact with other individuals during pairing and reproduction. With its shallow pits, the tegument of *P*. *homoion* resembles that of some other monogenean species, e.g. *Allodiscocotyla diacanthi* [46], *Empleurosoma pyriforme* [47] and *Eudiplozoon nipponicum* [16]. Similar to *E*. *nipponicum* [16,17], but unlike *Paranaella luquei* [48], *Marcorgyrodactylus congolensis* [49] or *Diclidophora merlangi* [50], we observed no microvilli or microvilli-like projections on the tegument surface of *P*. *homoion*.

Diplozoids have a number of superficially located sensory structures responsible for the reception and evaluation of information from the external environment (e.g. water flow), facilitating selection of suitable attachment sites on the surface of the host [2]. We showed that the well-innervated sensory structures are distributed over the entire body surface, being more concentrated in the forebody and hindbody areas. Abundant uniciliated sensory structures surrounding the mouth opening of *P*. *homoion* are likely related to surface perception of host tissue during attachment and food intake (blood sucking). They could also function as tangoreceptors with a tactile function or rheoreceptors for perception of water current during the parasite’s orientation [2]. The ultrastructure of the *P*. *homoion* sensory cilia has been described previously [33]. A comparison of the *P*. *homoion* cilium anchoring (shown in Fig 7E) with tangoreceptor (sensory structure with a single cilium) reconstructions for *Gyrodactylus* sp. and *Entobdella soleae* [51] indicates a resemblance with the striated transitional fibres arising from the basal body of the cilium that extend to the dense collar of the nerve bulb. Double fluorescent labelling confirmed that the rim of the sensory structures, clearly visible under SEM (Fig 7E), is rich in actin microfilaments (Fig 7H). This area corresponds to the septate desmosome [51] enveloping the nerve bulb and attached to the syncytium, which is likely to be F-actin rich in the same way as the septate junction previously described for invertebrates [52]. The fixed, long and movable cilium arising from the centre of the rim contained a high concertation of α-tubulin, a basic protein forming the microtubules [53]. We assume that the F-actin-rich peripheral rim may help position the cilium through constriction and dilation. This type of uniciliated sensory structures is similar to those observed in other monogeneans [46,51,54–56]. Moreover, the type of sensory structure in *P*. *homoion* corresponds to those surrounding the mouth area, though the rim appears more massive.

In addition to the uniciliated sensory structures, the apical part of the forebody has two round projections (Fig 1B) similar to those observed in *E*. *nipponicum* [16]. Using CLSM, we observed two circular, F-actin-rich structures (Fig 4A and 4B) similar to those observed in diporpa and other stages of *E*. *nipponicum* [17]. In *E*. *nipponicum*, two nerve cords terminated in this area [25], while our study showed dense innervation of the apex, likely related to the round projections. Hence, we speculate that these two projections could function as non-ciliated papillae [2, 51]. Phalloidin labelling revealed the presence of thin trabeculae oriented longitudinally towards the apex, the space in between being packed with nuclei, most likely belonging to the secretory cells. This suggests that some of the circular structures could be involved in secretion of substances involved in host-parasite interaction. This hypothesis is supported by a staining with hydrochloric carmine for CLSM revealing the presence of the putative gland cells accumulating in the region of apical circular structures. Similar gland cells were described in other monogeneans as adhesive glands [2]. Goto in his original description of *E*. *nipponicum* termed these as gigantic cells [5]. Another accumulation of gland-like cells in the middle region of *P*. *homoion* haptor may be also associated with adhesive function as proposed earlier [2].

The paired bowl-shaped buccal suckers in the mouth cavity appear to be the main attachment organs of the forebody. These suckers also help in sucking the host’s blood. As also observed in *E*. *nipponicum* [17], the U-shaped septum is located in the middle of the suckers. This structure is rich in F-actin and α-tubulin, and appears to be related to sucker innervation. Sucker functioning is controlled by prominent muscles that are directed around the pharynx posteriorly, and muscles that cross each other and are directed towards the apical part of the forebody. The muscles controlling the suckers are similar to those of *E*. *nipponicum* [17], except for the previously unobserved muscles fascicles oriented obliquely between the suckers.

The buccal cavity surrounding the suckers is covered with numerous foliate and tubular digitations up to the rim of the mouth, thereby expanding the parasite’s surface in contact with the host tissue. These tubular digitations have previously been described for *P*. *homoion* as short, irregular lamellae [33]. Similar projections were also recorded in the buccal cavity of *E*. *nipponicum* [16]. These digitations may enable insertion of biochemically active compounds for modifying host tissue during sucking, or could interact with the host’s blood before digestion. We expect the prominent gland-like cells surrounding the retractable pharynx in whole-mount preparations stained with hydrochloric carmine to be responsible for enzymatic secretion, with the paired club-shaped sacs opening towards the (pre)pharyngeal region and serving as secretory reservoirs.

The well-developed pharynx musculature and muscles controlling eversion and protruding of the pharynx correspond with those previously described in *E*. *nipponicum* [16,17]. The stretched muscles with deeply retracted pharynx (Fig 4C) and relaxed muscles with clearly shortened pharynx (Fig 4D) confirm the retractile function of these muscles. In general, the ultrastructure of the pharynx [33] corresponds with our CLSM observations. The four F-actin-rich circular openings detected on the apical end of the pharynx (corresponding with the pits in *E*. *nipponicum* visible under SEM ([17], Fig 6D), could represent ducts for releasing proteolytic enzymes during extraintestinal digestion, as proposed by Valigurová *et al*. [17]. As described in previous ultrastructural studies, these secretions could be produced by gland cells opening into the pharynx lumen [6,33].

As in other Platyhelminthes [17,57,58], the body wall musculature is organised into three main layers, an outer circular layer, an inner longitudinal layer and diagonal muscles. In *P*. *homoion*, additional muscles of unknown function run perpendicularly to the tegument, apparently corresponding to the dorso-ventral fibres reported in other studies [57]. Adult stages of *P*. *homoion* exhibit no obvious differences when compared to *E*. *nipponicum*; except for globular glandulo-muscular organs that appear to be species-specific for both the juvenile and adult stages of *E*. *nipponicum* [17].

Basic diagram showing the nervous system in *E*. *nipponicum* was published by Goto [5]. Later immunomicroscopical study focused on changes and fusion of central nerve elements in paired individuals of *E*. *nipponicum*, using a range of staining techniques to demonstrate cholinergic elements and FaRPergic innervation, along with serotonin immunostaining and gold labelling highlighting neuropeptide immunoreactivity [25]. Another study visualised the innervation of diporpa of *P*. *ichtyoxanthon* [37]. In our study, in addition to visualising the longitudinal nerve cords and transverse connectives of the central nervous system, immunofluorescent labelling of α-tubulin proved helpful in detecting a network of peripheral nerves reaching up to the tegumentary folds. Furthermore, this relatively simple method also helped visualise the fine innervation of sensory structures and their cilia. Nevertheless, the staining techniques used by Zurawski [25] do provide more specific results when identifying individual elements of the diplozoid nervous system.

Increased staining in the *P*. *homoion* apex indicates a higher accumulation of sensory structures and higher sensitivity of the entire mouth area. A similar combination of F-actin and tubulin staining was used to visualise musculature and innervation in a previous work on *Schistosoma mansoni* cercariae [40], though the antibody used detected β-tubulin. While the cephalic ganglia and main nerve cords were clearly stained for synapsin, β-tubulin was restricted to fine muscle innervation, especially in the caudal cercaria area, which was not labelled with synapsin. The antibody specific for acetylated α-tubulin was used in the above mentioned study to show the distribution of sensory papillae and the periphery of the acetabular glands.

Anti-α-tubulin antibodies were also used to localise *P*. *homoion* flame cells. Flame cells form part of the protonephridial system, which comprises a terminal (flame cell), an adjacent canal cell and a system of associated collecting ducts serving for osmoregulation, as previously described for other flatworms [44]. In selected monogenean species, previous studies focused on general organisation [5] and ultrastructure of the excretory system (e.g. [34,59]). Various isoforms of tubulin were used for investigation of the protonephridial system of *S*. *mansoni* [40]. Unfortunately, this method also visualises part of the nervous system. In order to avoid this cross-reaction, the authors used proteinase K to suppress nerve staining. The flame cell tuft roots were subsequently labelled with anti-phospho S/T antibody and DAPI, while the basket-like structure of the barrel (the non-ciliated part of the flame cell consisting of both the terminal and the adjacent canal cells) were stained with anti-phospho tyrosine and phalloidin. In our study on *P*. *homoion*, we achieved good results by using double fluorescent labelling, which stained the ciliated tuft (i.e. the ‘flame’) and cilia root for α-tubulin and the barrel for F-actin. The nucleus of the flame cell was easily detected using Hoechst counterstaining. While it is likely that the collecting ducts were also visualised using α-tubulin labelling (similar to a study on *S*. *mansoni* [40]), it was impossible to distinguish them reliably from the stained parts of the peripheral nervous system. Labelling of flame cells for β-tubulin in *S*. *mansoni* [40] corresponded to our results using the anti-α-tubulin antibody.

The hindbody of *P*. *homoion* plays an important role in attachment to host tissue and moving on the host’s gills. Compared to *E*. *nipponicum*, where prominent folds and species-typical lobular extensions play an important role in attaching this robust parasite within the gill lamellae [16,17], *P*. *homoion* has less prominent tegumentary folds and three distinct lobes on the haptor rather than lobular extensions. These lobes are highly mobile and equipped with a conspicuous three-layer musculature innervated with abundant uniciliated sensory structures on the surface. Besides the clamps and putative adhesive glands, these lobes serve presumably for attaching the parasite to the host’s gills. In contrast to *E*. *nipponicum*, in which the lateral hindbody is equipped with non-ciliated papillae (most likely involved in reception of environmental stimuli) [16], *P*. *homoion* had similar non-ciliated papillae clustered above the clamps. Such non-ciliated papillae have also been reported in *Entobdella soleae* [60,61]. It is generally assumed that these function as mechanoreceptors in direct contact with the host, with information from host-parasite interaction being utilised during attachment/detachment when relocating on the host’s surface [2]. Alternatively, they could serve as proprioreceptors for sensing the relative position of the haptor during movement.

As the primary structures fixing the parasite to the host surface, the clamps are organised in a similar manner to those in other Diplozoidae; i.e. four pairs of clamps in two parallel rows. While the sclerotised parts of the clamps exhibit autofluorescence and also are easily recognised under a light microscope equipped with Nomarski differential interference-contrast (NDIC; Fig 9G), the staining with Gomori trichrome appears to be the most suitable method for 3D visualisation of the sclerites and the marginal hooks [42]. Similar results were achieved in *Paradiplozoon* sp. using the Hörens trichrome [36]. Phalloidin staining confirmed that the clamp musculature, which is well developed and robust, is controlled by muscle bundles, which corresponds with previous observations on *E*. *nipponicum* [17,25] and *Diplozoon paradoxum* [6]. This musculature system controls individual clamps and facilitates parallel movement of an entire row of four clamps when translocating on host gill lamellae. In contrast to the visualisation of peptidergic and serotoninergic parts innervating the main muscles controlling the clamps in *E*. *nipponicum* [25], α-tubulin labelling in our study not only revealed innervation of the clamps but also the nerve fibres lining individual sclerites.

In conclusion, this study demonstrated the major structures important for the ectoparasitic life style of *P*. *homoion*. Overall, *P*. *homoion* exhibits a number of sophisticated functional adaptations to its ectoparasitic life-style, similar to those previously described for other helminth parasites (e.g. [9,17]). The original combined fluorescent labelling and SEM used in this study, however, revealed much more details in organisation and morphology of individual structures. The well-developed musculature and innervation of buccal suckers and haptor equipped with sclerotised clamps indicate their significant role in attachment and movement on the host. The hydrochloric carmine staining confirmed the increased accumulation of gland cells with proposed adhesive function in these regions [2]. The parasite is well equipped for blood sucking thanks to its heavily innervated mouth opening with abundant sensory structures, buccal cavity covered with numerous digitations, muscular buccal suckers and retractable pharynx with F-actin-rich circular openings on its apical end (likely representing the ducts for releasing proteolytic enzymes during extraintestinal digestion). On the top of that, we showed the presence of putative unicellular glands surrounding the pharynx along with so far not reported pair of club-shaped sacs that might function as secretory reservoirs. Using the fluorescent labelling, we were able to visualise the body wall musculature along with its peripheral innervation reaching up to the tegumentary folds, the distribution and innervation of uniciliated sensory structures and flame cells involved in parasite’s excretion.

## Abbreviations

AbD: antibody diluent
CLSM: confocal laser scanning microscopy
FITC: fluorescein isothiocyanate
IFA: α-tubulin immunofluorescence
F-actin: filamentous actin
PBS: phosphate buffered saline
SEM: scanning electron microscopy
TRITC: tetramethylrhodamine isothiocyanate
